# Evaluation of a Japanese brief CBT-I administered by a nurse: a pilot study

**DOI:** 10.1017/S1463423622000032

**Published:** 2022-08-03

**Authors:** Makie Nagai, Yuki Oe, Masaru Horikoshi, Shun Nakajima, Hitomi Oi, Yoshikuni Kita

**Affiliations:** 1 Tsuruga Nursing University, Tsuruga, Japan; 2 Department of Neuropsychiatry, Kyorin University, School of Medicine, Mitaka, Japan; 3 National Center for Cognitive Behavior Therapy and Research, National Center of Neurology and Psychiatry, Kodaira, Japan

**Keywords:** brief cognitive behavioral therapy, insomnia, pilot study, nurse

## Abstract

**Aim:**

The aim of this pilot study is to evaluate a Japanese version of brief Cognitive Behavioral Therapy for Insomnia (CBT-I) and contribute to primary care which leads to prevention of a lifestyle-related disease or a psychiatric disorder.

**Method:**

A single-arm study in nine patients with chronic insomnia who were under the pharmacotherapy was executed. The Insomnia Severity Index (ISI), the Athens Insomnia Scale (AIS), and the European Quality of Life 5 Items (EQ-5D) were assessed at the beginning of intervention, at the end of intervention, and after 12 weeks.

**Findings:**

There were no patient dropouts nor adverse events. The average change in ISI score was −7.33 (95% CI: −10.31 to −4.36) at post-treatment and −6.11 (95% CI: −8.20 to −4.03) at the 12-week follow-up point (Cohen’s d = 2.25). The AIS score improved as well, and the EQ-5D score improved after 12 weeks. The safety and efficacy of the brief CBT-I were suggested.

## Introduction

In Japan, about one in five people complain of sleep difficulty at night (Kim *et al.*, 2000). The effects of disrupted sleep are not limited to disrupting social life but have also become important factors in lifestyle-related diseases (Gangwisch *et al.*, 2006; Gottlieb *et al.*, 2006; Hall *et al.*, 2008). The impacts of insomnia are also associated with mental health challenges such as depression, anxiety disorders, and substance dependence (Gillin, 1998; Ohayon and Roth, 2003). Improving insomnia is important primary care which leads to prevention of a lifestyle-related disease or a psychiatric disorder.

Hypnotic drugs are used to treat insomnia in 1 out of 20 people in Japan (Kaneita *et al.*, 2007). However, the European Guidelines for the Diagnosis and Treatment of Insomnia recommend medication when the effects of Cognitive Behavioral Therapy for Insomnia (CBT-I) are not sufficient or it cannot be used (Riemann *et al.*, 2017). In addition, the 10 min CBT-I (brief CBT-I) was developed in the UK (David, 2006), and the effects of brief CBT-I have begun to be reported (Palermo *et al.*, 2017). They reported that brief CBT-I was associated with improvements in self-reported measures of sleep, including insomnia symptoms, sleep quality, sleep hygiene, pre-sleep arousal, and sleep onset latency, and that the effects generally persisted at 3-month follow-up. If a nurse can perform brief CBT-I widely at the medical clinic in Japan, it leads to CBT-I spread and can expect development of primary care. In this pilot study, we report the positive results from the brief CBT-I administered by a trained nurse in Japan.

## Material and methods

### Participants

The present clinical trial was conducted as a single-arm study. There were nine study candidates recruited from the psychiatric clinic and hospital outpatients. The age range was set at 20 years or greater and subjects were selected regardless of sex. The inclusion and exclusion criteria were established for candidate selection, and the family doctors (research colleagues) of the patients were asked to identify study participants based on these criteria.

The inclusion criteria were as follows: (1) the current pattern of insomnia met the diagnostic criteria for a chronic insomnia obstacle (3rd edition of the Sleep Disorder International Classification) (The Japanese Society of Sleep Research, 2018); (2) the Insomnia Severity Index (ISI) score was ten or more points; and (3) a specific hypnotic drug was currently being prescribed and used. The exclusion criteria were as follows: (1) presence of serious physical pain, such as sharp pains, or breathing difficulties; (2) diagnosis of dementia, manic state, schizophrenia, or substance dependence (American Psychiatric Association, 2013); (3) a Beck Depression Inventory-II score of 31 or more points (Beck *et al.*, 1996); and (4) candidates with complicated social backgrounds, such as family violence.

### Intervention and procedure

The brief CBT-I was based on information available from previous studies conducted outside of Japan (David, 2006; Palermo *et al.*, 2017). The protocol for the brief CBT-I consisted of a maximum of four sessions, with each being about 10 min in length.

**Figure 1.**
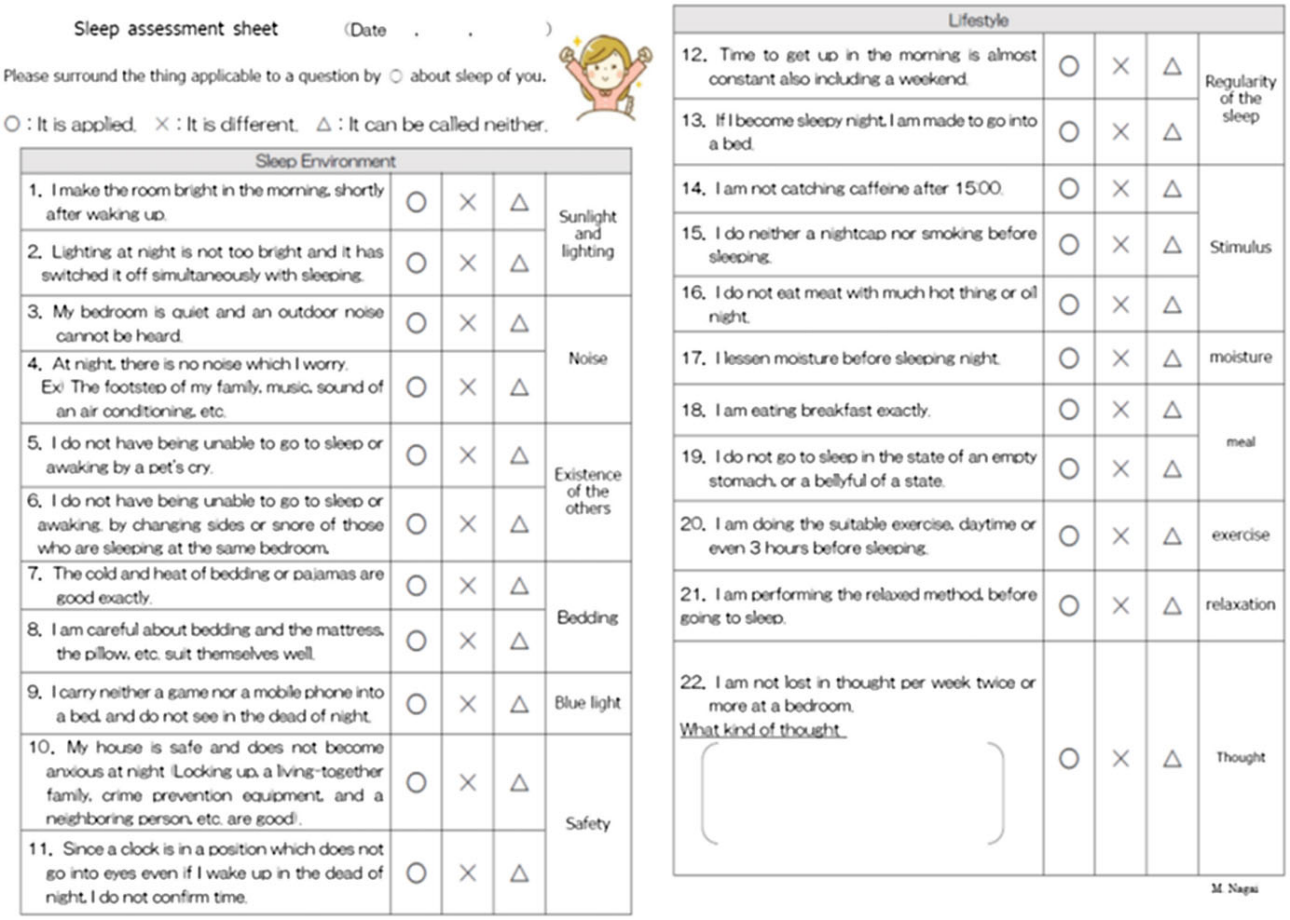
Sleep assessment sheet.

**Figure 2.**
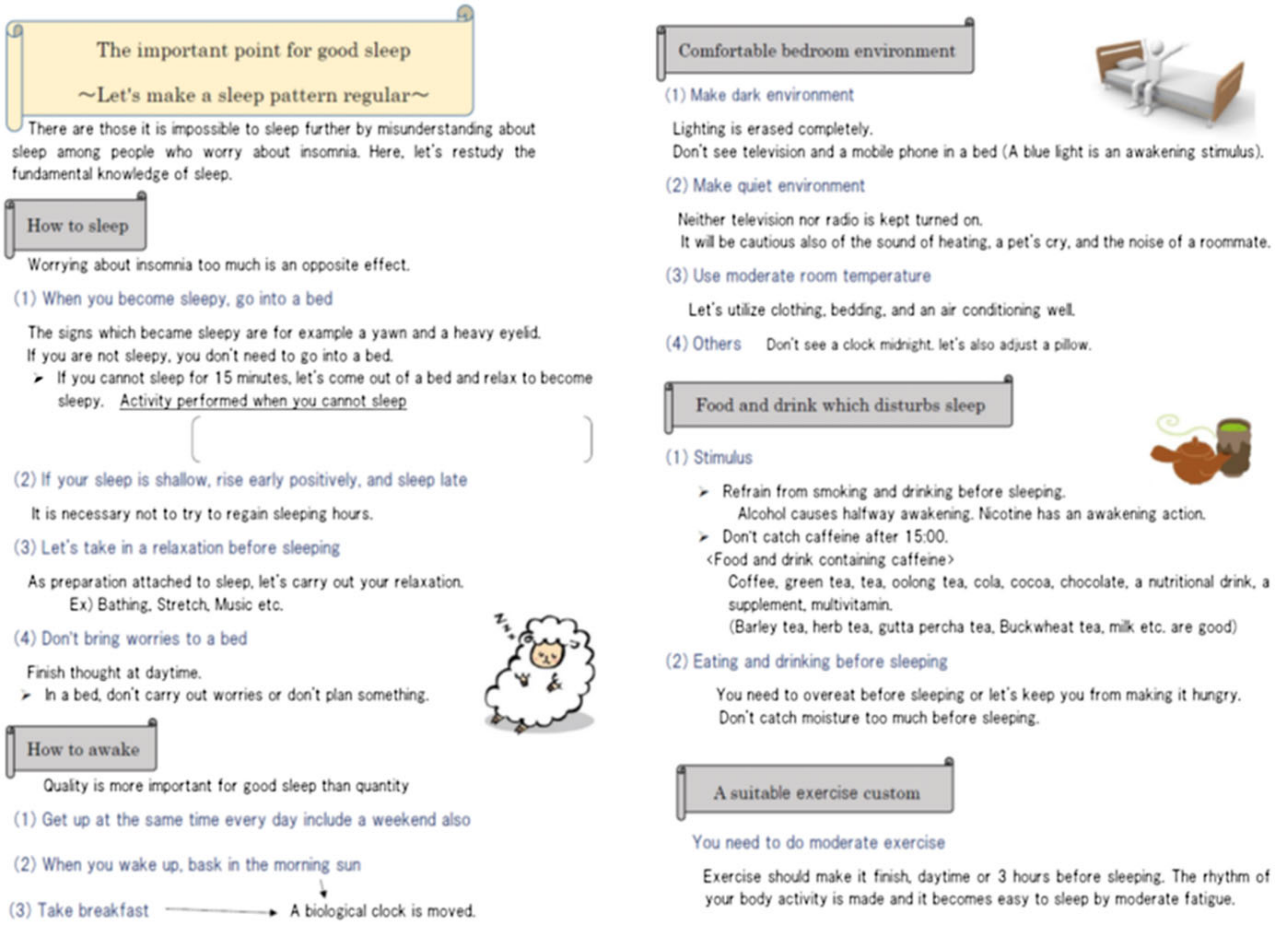
Educational sheet.

The topic and contents of each session were clearly defined. In the first session, the patient described their main issues pertaining to insomnia with the nurse, and the nurse explained the homework of completing a sleep assessment sheet and a sleep diary (Figure 1). In the second session, the nurse examined the homework with the patient and provided psychoeducation (Figure 2). The psychoeducation focused only on points identified as problematic based on the sleep assessment sheet and included the use of sleep hygiene, cognitive therapy, and the sleep scheduling method. The patient was then advised to practice the psychoeducation methods at home and a sleep diary was assigned as homework. The third session was performed similarly to the second session. In the fourth session, the patient was asked to consider how to prevent the recurrence of insomnia by trying to imagine the types of situations that might worsen the problem, and how to manage these situations. Instructional material for sleep hygiene was also provided.

After receiving an introduction from the family doctor, the nurse interviewed participants in order to explain the study’s purpose and content. The nurse ensured that there was complete understanding and cooperation in the research, then the first brief CBT-I session was started after obtaining consent. Brief CBT-I was provided weekly or biweekly at the psychiatric outpatient clinic and hospital.

### Training for the nurse and maintenance of technical quality

The nurse that administered this pilot study completed formal cognitive behavior therapy training (sponsored by the Ministry of Health, Labor and Welfare, etc.) for a total of 100 h or more, and practiced administering CBT to ten patients for depression and anxiety (including 2 cases supervised by the most senior instructor).

In order to maintain the technical quality of the brief CBT-I, enrollment was limited to five concurrent patients. The nurse reviewed every session using either audio recordings obtained during each session or notes which were taken if the participant did not permit audio recording.

### Measures and procedures

Fundamental information for each patient was collected, including age, sex, the identity of the main disease, complications, the highest level of education completed, and the type and quantity of hypnotic used. The severity of insomnia was set as the primary endpoint of this study. It was assessed using self-report questionnaires of the ISI and the Athens Insomnia Scale (AIS). Secondary endpoint pertaining to quality of life (QOL) was assessed using the European Health-Related Quality of Life. All measurement tools totaled 20 items written in Japanese. Data collection carried out prior to the beginning of brief CBT-I (week 0), at the end of treatment (4 weeks), and at the follow-up point (12 weeks).

In addition, in order to suitably evaluate the intervention, the family doctors were requested no change in medication type or dosage.

#### The ISI

ISI is a measure that reflects the previous 2 weeks and subjectively evaluates the insomnia severity of illness. The ISI measures the nature, severity, and adverse effects of insomnia based on scores from a 7-item questionnaire (5-point Likert scale, 0 = no problem, 4 = very severe problem). The total score (range 0–28) is interpreted as follows: 0–7, no clinically significant insomnia; 8–14, sub-threshold insomnia; 15–21, moderate insomnia; and 22–28, severe insomnia. The Japanese version of the ISI has a high internal consistency (α = 0.84), and its reliability and validity have been verified (Munesawa *et al.*, 2009). The cut-off value was 10 points or more.

#### The AIS

The AIS is a measure which reflects back upon the previous 1 month and evaluates the quality of sleep. It consists of five items relating to nocturnal sleep problems and three items relating to daytime dysfunction. Each item is scored using a 4-point Likert scale (0 = no problem at all, 3 = extremely problematic). The Japanese version has excellent internal consistency (Cronbach’s alpha values = 0.78–0.88), and concurrent validity that the correlation between the AIS and Pittsburgh Sleep Quality Index was significantly high (r = 0.81, 95% CI: 0.82–0.87) (Okajima *et al.*, 2013a). The cut-off value was 6 points or more.

#### The European Quality of Life 5 items (EQ-5D)

The EQ-5D is a comprehensive tool used in every country in the world and measures health-related QOL (HRQOL). The reliability and validity of the Japanese version of the HRQOL have been previously verified (Nishimura *et al.*, 1998). The Japanese EQ-5D consists of a 5-item descriptive questionnaire of movement, management of personal appearance, activity, pain, and displeasure and uneasiness. It also includes a visual appraisal method which asks the subjects to freely draw their present health condition using a line. We converted the HRQOL score to the health condition acquired from the 5-item method using the conversion table.

### Statistical analysis

The feasibility of using the Japanese brief CBT-I was evaluated based on the rate of dropouts and adverse events.

For all data obtained from the AIS, ISI, and EQ-5D, we calculated the amount of change after treatment and at the 12-week follow-up. After assessing the distribution of the amount of change, the values at baseline and post-treatment as well as the values at baseline and the 12-week follow-up were compared. The effect size for the mean difference at the follow-up point was computed using Cohen’s d (G * Power).

For secondary analysis, the improvement rate at post-treatment and at the 12-week follow-up point was calculated using the respective cut-off points (ISI score = 10.0, AIS score = 6.0).

In this study, the significance level was set at *P* < 0.05. All statistical analyses were conducted using SPSS version 23.0 for Windows (IBM, Tokyo, Japan).

## Results

### Participant characteristics

Characteristic data were obtained from the nine participants. The mean age was 55.0 years [standard deviation (SD) = 18.3, range = 22–75] and 66.6% were female. Depression and unidentifiable depression per person existed, respectively. All patients were currently taking some hypnotic drugs.

### Dropout and adverse events

In this clinical trial, there were no patient dropouts nor adverse events. The session time of the brief CBT-I required on average 12.8 min (range = 5–22 min).

### Changes in insomnia measurements

**Figure 3.**
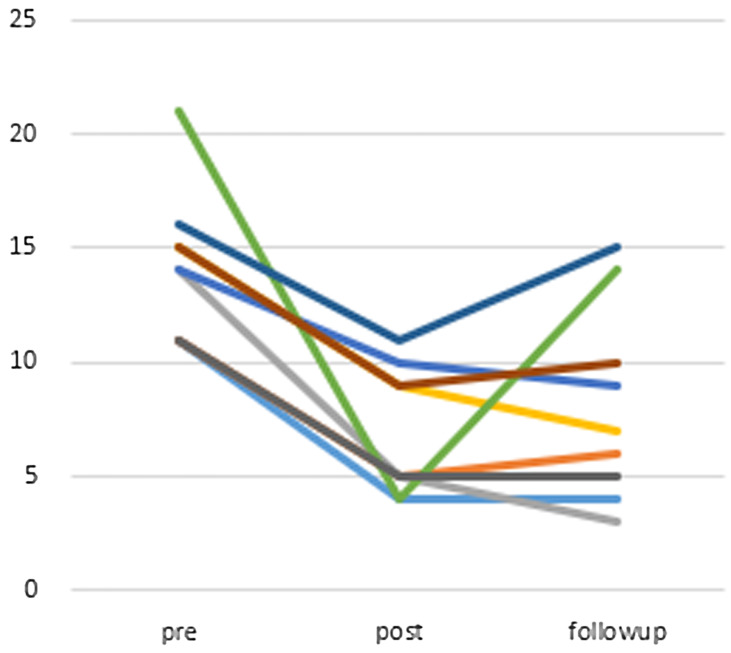
Changes of points of ISI (compared to post-treatment *P* = 0.007, compared to follow-up (12 weeks after) *P* = 0.007 (Wilcoxon signed-rank test)), *n* = 9.

The average change in ISI score was −7.33 (95% CI: −10.31 to −4.36) at post-treatment and −6.11 (95% CI: −8.20 to −4.03) at the 12-week follow-up point (Figure 3). The ISI score improved significantly when comparing baseline with post-treatment (Wilcoxon signed-rank test, *P* = 0.007), and baseline with the 12-week follow-up point (Wilcoxon signed-rank test, *P* = 0.007). The effect size for change in ISI scores from baseline to the follow-up point (Cohen’s d = 2.25) was high.

**Figure 4.**
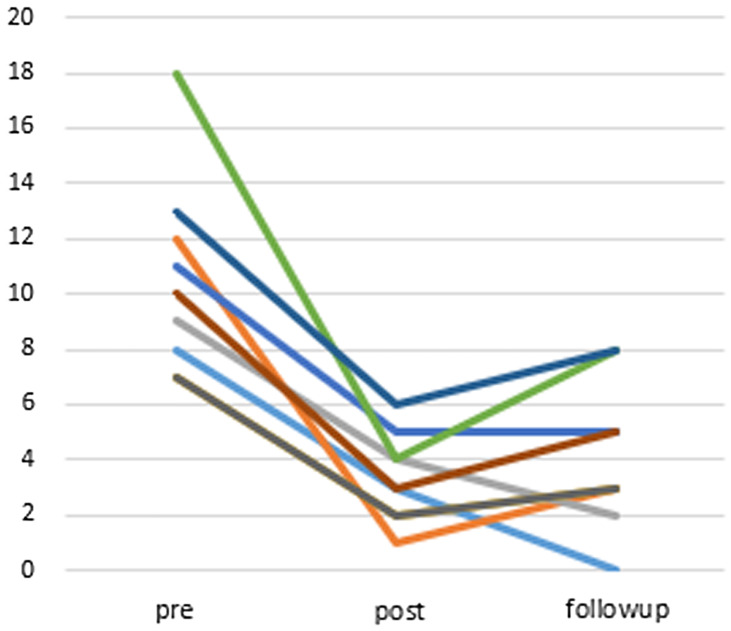
Changes of points of AIS-J (compared to post-treatment *P* = 0.007, compared to follow-up (12 weeks after) *P* = 0.008 (Wilcoxon signed-rank test)), *n* = 9.

The average change in the AIS-J score was −7.22 (95% CI: −9.68 to −4.77) at post-treatment and −6.44 (95% CI: −8.12 to −4.76) at the 12-week follow-up point (Figure 4). The AIS-J score also improved significantly when comparing the baseline with post-treatment (Wilcoxon signed-rank test, *P* = 0.007), as well as the baseline with the 12-week follow-up point (Wilcoxon signed-rank test, *P* = 0.008). The effect size for change in AIS-J scores from baseline to the follow-up point (Cohen’s d = 2.95) was high.

### Changes in QOL

**Figure 5.**
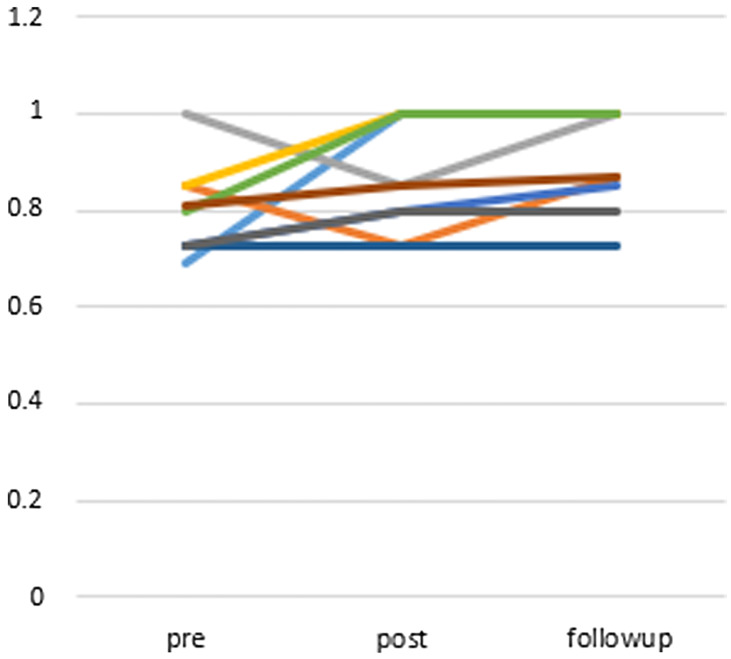
Changes of points of EQ-5D (compared to post-treatment *P* = 0.233, compared to follow-up (12 weeks after) *P* = 0.018 (Wilcoxon signed-rank test)), *n* = 9.

Figure 5 shows the graphed score change in the EQ-5D. The average change in the EQ-5D score was 0.06 (95% CI: −0.05 to 0.18) at post-treatment and 0.10 (95% CI: 0.02 to 0.18) at the follow-up point. The EQ-5D score showed mild improvement when comparing scores at baseline with post-treatment (Wilcoxon signed-rank test, *P* = 0.233) but showed significant improvement when comparing baseline with the 12-week follow-up scores (Wilcoxon signed-rank test, *P* = 0.018). The effect size for change in EQ-5D scores from baseline to the follow-up point (Cohen’s d = 0.99) was large.

### Improvement in the ate of insomnia

The improvement rate in the ISI scores calculated at post-treatment was 77.8% (7 subjects) and at the follow-up point was 66.7% (6 subjects). The improvement rate in the AIS-J score calculated at post-treatment was 88.9% (8 subjects) and at follow-up was 77.8% (7 subjects). At the end of the brief CBT-I, one person stopped using the hypnotic and one person decreased the quantity of the hypnotic used. There were no other changes in drug prescriptions.

## Discussion

Neither an adverse event nor a dropout was seen by brief CBT-I developed in this research. It is meaningful toward the next research that RCT of Japanese brief CBT-I. If a nurse can perform brief CBT-I widely at the clinic in Japan, it leads to prevention of a lifestyle-related disease or a psychiatric disorder and can expect development of primary care. This is because the brief CBT-I by nurse is expected to make the treatment of sleep more effective through the patient’s own self-care rather than through medication.

The improvement rate was also equivalent to the remission rate of standard CBT-I (71%) for medicated insomnia in Japan (Okajima *et al.*, 2013b) and the remission rate at the 12-week follow-up was also maintained. The following effect sizes of brief CBT-I for changes in ISI scores may be comparable with other versions of CBT-I or brief CBT-I (Palermo *et al.*, 2017; Okajima and Inoue, 2018). However, these results are limited in interpretation given that there were only nine study participants.

The average time required to administer the brief CBT-I used in this study was 12.8 min. Traditional CBT-I includes all elements in each assessment, such as sleep hygiene, stimulus control therapy, sleep restriction therapy, cognitive therapy, and relaxation (Smith *et al.*, 2002; Okajima *et al.*, 2011; Trauer *et al.*, 2015; Wu *et al.*, 2015). However, the factors which cause insomnia are varied and thus many of these items may be unrelated to the experience of the patient. Here, we aimed to shorten the time required to administer the brief CBT-I by focusing on the main factors associated with an individual patient’s insomnia. The brief CBT-I was designed to fit on one sheet and focused on key points compared to the standard CBT-I which advances along with a text. Only content relevant to each participant was circled and then those items were explained in detail. Therefore, only the necessary minimal information was provided to the participant, allowing them to focus on their homework. Thus, the educational materials, psychoeducation, and homework are all important components of this study.

## Conclusion

This pilot study was aimed at developing a Japanese version of brief CBT-I, and results suggested the safety and efficacy of this treatment. In order to offer the primary care for insomnia improvement at the medical clinic in Japan, it is necessary to carry out RCT of the brief CBT-I which can be administered by a nurse.
